# Detection of human coronaviruses in simultaneously collected stool samples and nasopharyngeal swabs from hospitalized children with acute gastroenteritis

**DOI:** 10.1186/1743-422X-10-46

**Published:** 2013-02-05

**Authors:** Monika Jevšnik, Andrej Steyer, Tamara Zrim, Marko Pokorn, Tatjana Mrvič, Štefan Grosek, Franc Strle, Lara Lusa, Miroslav Petrovec

**Affiliations:** 1 Institute of Microbiology and Immunology, Faculty of Medicine, University of Ljubljana, Zaloška 4, 1000 Ljubljana, Slovenia; 2 Department of Infectious Diseases, University Medical Centre Ljubljana, Japljeva 2, 1525 Ljubljana, Slovenia; 3 Department of Pediatric Surgery and Intensive Care, University Medical Centre Ljubljana, Bohoričeva 20, 1000 Ljubljana, Slovenia; 4 Institute of Biostatistics and Medical Informatics, Faculty of Medicine, Vrazov trg 2, 1104 Ljubljana, Slovenia

**Keywords:** Human coronavirus, Viral gastroenteritis, Respiratory infection

## Abstract

**Background:**

Human coronaviruses (HCoVs) are a well-known cause of respiratory infections but their role in gastrointestinal infections is unclear. The objective of our study was to assess the significance of HCoVs in the etiology of acute gastroenteritis (AGE) in children <6 years of age.

**Methods:**

Stool samples and nasopharyngeal (NP) swabs collected from 260 children hospitalized for AGE (160 also had respiratory symptoms) and 157 otherwise healthy control children admitted for elective surgery were tested for the presence of four HCoVs using real time RT-PCR. Registered at ClinicalTrials.gov (reg. NCT00987519).

**Results:**

HCoVs were more frequent in patients with AGE than in controls (23/260, 8.8% versus 4/151, 2.6%; odds ratio, OR 3.3; 95% confidence interval, CI 1.3–10.0; *P* = 0.01). Three of four HCoV-positive members in the control group, asymptomatic when sampled, recalled gastrointestinal or respiratory symptoms within the previous 14 days. In patients with AGE, HCoVs were present in NP samples more often than in stools (22/256, 8.6%, versus 6/260, 2.3%; *P* = 0.0004). In 5/6 children with HCoVs detected in stools, the viruses were also detected in NP swabs. Patients had a significantly higher probability of HCoV detection in stool (OR 4; 95% CI 1.4–15.3; *P* = 0.006) and also in stool and/or NP (OR 3.3, 95% CI 1.3–10.0; *P* = 0.01) than healthy controls. All four HCoVs species were detected in stool and NP samples.

**Conclusions:**

Although HCoVs were more frequently detected in patients with AGE than in the control group, high prevalence of HCoVs in NP swabs compounded by their low occurrence in stool samples and detection of other viruses in stool samples, indicate that HCoVs probably play only a minor role in causing gastrointestinal illness in children <6 years old.

## Background

Human coronaviruses (HCoVs), known since the late 1960s, have been recognized as a frequent cause of mild respiratory tract infections (RTIs) and occasionally as a potential cause of severe lower RTI in premature infants and children with underlying diseases [1]. Their role in enteric infections is less clear. Even though coronavirus-like particles have been seen by electron microscopy in stool samples from patients with diarrhea, they have been also found in healthy individuals [2]. However, coronaviruses are associated with diarrheal disease in several animal species. Thus, a dual enteric and respiratory tropism has been reported for the bovine coronavirus causing winter dysentery in calves [3]. Interest in coronaviruses in relation to enteric diseases in humans increased with the emergence of severe acute respiratory syndrome (SARS) and identification of SARS-CoV in 2003 [4,5]. SARS was characterized clinically by signs/symptoms of severe lower RTI but several patients also had gastrointestinal complaints [6,7]. Diarrhea has also been a prominent clinical feature in several patients with respiratory infection caused by other HCoVs [8-10]. Furthermore, all HCoV species were recently detected in stool samples of patients with acute gastroenteritis (AGE) by modern molecular methods, but their role in human gastrointestinal infections remains uncertain [11-13]. In previous studies, HCoVs were demonstrated in patients with gastrointestinal and/or respiratory involvement, but in none of these studies was concurrent collection of stool and respiratory samples performed. The main objective of the present study was to evaluate the presence of HCoVs in simultaneously collected stool samples and nasopharyngeal (NP) swabs in children with AGE (with or without associated respiratory symptoms) and in control subjects, with the aim of appraising their role in the etiology of AGE.

## Results

### Patients

From October 2009 to September 2011, 765 children <6 years of age were referred to the Department of Infectious Disease with the diagnosis of AGE. Of them 260 were enrolled in the study. The others did not meet inclusion criteria for AGE (250 children), were not enrolled because their parents did not consent with the inclusion in the study (182 children) or because they did not pass stool during hospital stay (73 children). The median age of included children was 18.9 months. The female: male subject ratio was 1: 1.13 (122/260; 46.9% girls). In 100 of the subjects (39.5%), signs and symptoms were limited to the gastrointestinal tract, while 160 (61.5%) also had signs and symptoms compatible with acute RTI such as rhinitis, pharyngitis, conjunctivitis, otitis or bronchiolitis.

In the group of children with AGE, both samples (stool sample and NP swab) were obtained from 256/260 patients (only stool sample was acquired from four of them). From 157 children comprising the healthy control group, both samples were available from 150 patients, only a stool sample from one child and only a NP swab from six.

Samples obtained at follow-up examination 14 days after initial testing were available for 192/260 (73.8%) children with AGE. Unfortunately, 68 remaining children did not respond and did not come to follow up visit 14 days after acute illness. Children who did not come to the follow up visit had similar clinical characteristics as those who attended the follow up visit. Therefore, authors assumed that they were representative of the entire study population. In 174 of the patients, both stool and NP swab samples were obtained, while only stool sample was obtained from nine children and solely NP swab was available from nine others.

### Detection of HCoVs in stool samples and NP swabs of patients with AGE

HCoVs were detected in 6/260 stool samples from children with AGE (2.3%; 95% CI 0.9–5.0%)—among which 5/6 were detected also in NP samples—and in 22/256 NP swabs (8.6%, 95% CI 5.5–12.7%). HCoVs were more often detected in NP swabs than in stool samples (*P* = 0.0004 by McNemar’s test).

Four of six (67%, 95% CI 22–96%) children with HCoV-positive stool samples had symptoms of respiratory and gastrointestinal infection (Table 1). Of the six HCoV-positive stool samples, three were characterized as OC43, one as NL63, one as HKU1 and one as 229E. In two (33.3%) stool samples HCoV (one 229E and one OC43) was detected as a single pathogen, while in four samples the presence of other viruses was also established (Table 2).

**Table 1:** RT–PCR positivity for HCoVs according to sample type and clinical presentation

	**HCoVs stool positive**	**HCoVs stool negative**	**HCoVs NP swab positive**	**HCoVs NP swab negative**	**Both stool and NP swab HCoVs positive**
Patients	6/260 (2.3%, 95% CI 0.9–5.0%)	254/260 (97.7%, 95% CI 95-99%)	22/256 (8.6%, 95% CI 5.5–12.7%)	234/256 (91.4%, CI 87-95% )	5/256 (1.9%, 95% CI 0.6-4.5%)
AGE only	2/6, (33%, 95% CI 4.3–78%)	98/254 (38.6%, 95% CI: 33-45% )	9/22 (41%, 95% CI 21–64%)	87/234 (3.2%, CI 31-44%)	1/5, (20%. 95% CI 0.5–72%)
AGE + RTI	4/6 (66.7%, 95% CI 22–96%)	156/254 (61.4%, 95% CI: 55-67%)	13/22 (59%, 95% CI 36–79%)	147/234 (62.8%, 95 CI 56-69%)	4/5, (80%, 95% CI 28–99%)
Vomiting	6/6 (100%, 95% CI 54-100%)	201/254 (79.1%, 95% CI 74-84%)	15/22 (68.2%, 95% CI 45-86%)	188/234 (80.3%, 95% CI 75-85%)	5/5 (100%, 95% CI 48-100% )
Median duration of illness (days)	5.5 (range 4–16)	6 (range 1–18)	5 (range 1–16)	6 (range 1–18)	5 (range 4–16)
Median duration of hospitalization (days)	3 (range 2–6)	2 (range 1–11)	2.5 (range 1–6)	2 (range 1–11)	3 (range 2–6)
Controls	1/151 (0.7%, 95% CI 0–3.6%)	150/151 (99.3%, 95% CI 96.4-100%)	3/150 (1.9%, 95% CI 0.4–5.7%)	147/150 (98%, 95% CI 94.3-99.6%)	0

Key: AGE, acute gastroenteritis; RTI, respiratory tract infection; NP, nasopharyngeal.

**Table 2:** Patients with HCoVs demonstrated in stool samples and/or NP swabs at initial testing: information on clinical signs, viral species and results of follow-up testing

**Patient**	**Clinical signs**	**HCoVs species in stool samples**	**Other viruses in stool samples**	**HCoVs species in NP swab**	**Other viruses in NP swab**	**Follow up stool sample**	**Follow up NP swab**
1	AGE + RTI	OC43	neg	OC43	neg	neg	HKU1*
2	AGE + RTI	OC43	Norovirus	OC43	neg	neg	neg
3	AGE + RTI	OC43	Rotavirus, AdV	OC43	AdV	AdV	OC43, AdV
4	AGE + RTI	NL63	Rotavirus	NL63	hRV	neg	NL63, hRV, PIV*
5	AGE	HKU1	SRV	OC43	HBoV, hRV, PIV	neg	HKU1**
6	AGE	229E	neg	neg	neg	neg	neg
7	AGE + RTI	neg	neg	OC43	neg	neg	neg
8	AGE + RTI	neg	neg	OC43	neg	NA	NA
9	AGE + RTI	neg	neg	NL63	hRV	neg	neg*
10	AGE + RTI	neg	Astrovirus	NL63	RSV, INF A	neg	neg
11	AGE + RTI	neg	Rotavirus	HKU1	neg	neg	RSV,PIV*
12	AGE + RTI	neg	Rotavirus	HKU1	neg	NA	NA
13	AGE + RTI	neg	AdV	HKU1	AdV, RSV	NA	NA
14	AGE + RTI	neg	Rotavirus, AdV	HKU1	neg	neg	hRV
15	AGE + RTI	neg	Norovirus, AdV	HKU1	AdV, HBoV	neg	neg
16	AGE	neg	neg	OC43	neg	neg	neg
17	AGE	neg	neg	229E	neg	neg	neg
18	AGE	neg	Rotavirus	OC43	neg	neg	neg
19	AGE	neg	Rotavirus, AdV	OC43	neg	neg	NA
20	AGE	neg	Rotavirus	HKU1	neg	neg	neg
21	AGE	neg	Norovirus, AdV	HKU1	neg	AdV**	HKU1
22	AGE	neg	Rotavirus, AdV	229E	neg	neg	RSV*
23	AGE	neg	Rotavirus	229E	neg	neg	neg

Key: *RTI, respiratory tract infection; **AGE, acute gastroenteritis; OC43; human coronavirus OC43; NL63, human coronavirus NL63; HKU1, human coronavirus HKU1; 229E, human coronavirus 229E; AdV, adenovirus; hRV, rinovirus; PIV, parainfluenza virus; SRV, small round viruses; RSV, respiratory syncytial virus; INF A, influenza virus A; neg, negative; NA, sample not available.

Of 22 positive NP samples, 13 (59%, 95% CI 36–79%) originated from children with AGE associated with RTI. The species distribution was as follows: nine OC43 (41%), seven HKU1 (32%), three 229E (14%) and three NL63 (14%; Table 2). Fifteen (68.2%) of 22 HCoVs positive NP swabs were detected as a single viral pathogen; also in this subgroup the most common was OC43 (7/15, 46.7%). HCoVs was detected as a single viral pathogen in NP swab in 8/9 (88.9%) children with AGE as the only clinical sign and in 7/13 (53.8%) NP swabs of children who had AGE associated with signs/symptoms of RTI (OR = 6.3; 95% CI 0.5- 353; *P =* 0.16).

HCoVs were not detected in any of 183 follow-up stool samples. Of 183 follow-up NP swabs, 15 (8.2%, 95% CI 4.7–13.1%) were positive for HCoVs: eight were typed as HKU1, four as NL63 and two as OC43; one positive sample remained untyped because of the low viral load. In 10 of 15 positive children, HCoVs were demonstrated only in the follow-up sample, while in five children the presence of HCoVs was established in the first and follow-up NP swab; four of these also had HCoVs in stools at the first sampling (Table 2). At the time of follow-up testing, 1/15 children (7%, 95% CI 0.2–31.9%) with HCoV-positive NP swab had gastrointestinal signs/symptoms; 10/15 (67%, 95% CI 38–88%) had RTI and four (26.7%, 95% CI 8–55%) were asymptomatic.

None of the 38 samples from 33 children positive for HCoVs in NP swabs and/or stool sample had viruses detected in blood sample taken simultaneously.

### Control group

HCoVs were detected in 1/151 stool samples (0.7%, 95% CI 0–3.6%) and in 3/150 NP swabs (1.9%, 95% CI 0.4–5.7%) from the control group. The HCoV from the stool sample remained untyped whereas respiratory samples revealed one NL63, one 229E and one HCoV that was untyped. HCoVs remained untyped because of the low viral load. All 151 children included in the control group were asymptomatic on the day of sampling; however a detailed history showed that three out of four children who were found to be HCoV positive reported symptoms indicating respiratory or gastrointestinal infections within 14 days before sampling.

Patients with AGE had a significantly higher probability of HCoV detection in stool samples than controls (OR 4; 95% CI 1.4–15.3; *P* = 0.006), while a weaker association was observed using NP samples (OR 2.6; 95% CI 0.5–24.7; *P* = 0.26). If positive stool samples and/or NP swabs were considered together, the association remained statistically significant (OR 3.3; 95% CI 1.3–10.0; *P* = 0.01).

## Discussion

HCoVs are recognized as causes of respiratory infections but their role in gastrointestinal infections has not been clarified. In the present study we assessed the significance of HCoVs in the etiology of AGE in children <6 years old by testing samples from stools and NP swabs for the presence of four HCoVs by RT–PCR. In the patients with AGE, follow-up sampling was performed 14 days after the initial testing and we acquired detailed clinical information for each participant. Our study revealed that HCoVs were present in patients with AGE and that viruses were more prevalent in the patients than in the control group (23/260, 8.8% versus 4/151, 2.6%, OR 3.3, 95% CI 1.3–10.0%, *P* = 0.01). Moreover, the higher probability of HCoV detection in the stool samples of patients with AGE than among controls (OR 4; 95% CI 1.4–15.3; *P* = 0.006) indicated a causal association of HCoVs with AGE. This was supported by the observation that three of four HCoV-positive members of the control group, who were asymptomatic at the time of sampling, reported symptoms within a 14-day period before sampling. However, in patients with AGE one would think that the etiologic agent is more likely to be demonstrated predominantly in stool samples, whereas in our study HCoVs were detected more often in NP swabs than in stools (22/256, 8.6% versus 6/260, 2.3%; *P* = 0.0004). In addition, in five of six children with HCoVs detected in the stool samples, the viruses were also demonstrated in NP swabs. There are several partial explanations for this. The course of the events in HCoVs infection might have been similar to those known to be operative in animals and as suggested for SARS-CoV [6,14]. Thus, fecal–oral transmission is followed by intestinal damage, leading to viremia and respiratory infection. However, we tested all patients with HCoVs found in stool samples or NP swabs for the presence of the viruses in blood but did not get any positive result. In addition, viremia usually results in the involvement of the lower RTI whereas upper RTI is usually the result of direct spread of infection through neighboring tissues. Although we cannot exclude viremia that was missed in our patients, it seems more likely that HCoVs did not cause viremia. The other possibility is that oral transmission of HCoVs is followed by gastrointestinal infection in patients with AGE and in the majority of children by symptomatic or asymptomatic NP infections because of the direct spread of viruses from the oral cavity to the nasopharynx, or secondarily from the gastrointestinal region from vomiting (69.6% of our children experienced vomiting in addition to diarrhea). Because enveloped HCoVs viruses are not stable in stools, their persistence is much more limited and transient than in the upper respiratory tract. This could explain the lower detection rate of HCoVs in stools than in NP swabs. The third possibility is that the primary location of HCoVs replication is in the nasopharynx, resulting in asymptomatic or symptomatic respiratory infections, and that the HCoVs occasionally found in stool samples had their origin in the respiratory system but were passed down the alimentary tract. This theory would enable an elegant explanation for the higher proportion of NP swabs positive for HCoVs than in stools and for the finding that all but one patient with HCoVs in the stool samples also had them present in NP swabs, but does not explain AGE, which should have been caused by other agent. The theory is further supported by the finding that only in two children HCoVs were found as a sole viral agent in stool while in four cases the presence of other viruses known to cause AGE was also established. Surprisingly, HCoVs were detected as a single viral pathogen in 8/9 (88.9%) NP swabs from children with AGE without concomitant respiratory symptoms.

More than half (160/260, 61.5%) of children with AGE also had respiratory symptoms at the time of sampling. This proportion was similar for patients with HCoV-positive NP swabs (59.1%) and for those with HCoV-positive stool samples (66.7%), whereas it was higher (80%) for children with HCoVs detected in both samples. However, there were very few patients with HCoVs in stool samples and with viruses found in both sources (six and five, respectively) and the difference was not statistically significant. In a report from Finland, 50% of children with AGE and HCoV-positive stool samples had respiratory symptoms at the time of sampling, while in a study from the USA both symptoms appeared in 75% of children [11,13]. The median duration of illness and of hospitalizations were similar for HCoVs positive and negative children (Table 1), and the differences were not statistically significant (duration of illness: *P* = 0.91 for stool samples, *P* = 0.63 for NP swabs, duration of hospitalization: *P* = 0.11 for stool samples and *P* = 0.13 for NP swabs).

No significant difference was found between the proportion of HCoVs present at the time of acute illness and 14 days later (23/260, 8.8%, versus 15/183, 8.2%, *P* = 0.54). A more detailed analysis of follow-up samples revealed that the presence of HCoVs in all 15 children was limited to NP swabs, that only 5/15 (33.3%) children had been positive for HCoVs in the first sample and that in 4/5 of them HCoVs were found in NP swabs as well as in stool samples. The last finding suggests that the persistence of HCoVs in NP swabs is associated with positivity of both samples (NP swabs and stools) during an acute illness.

Information on the occurrence of HCoVs in healthy persons is limited. Our finding that HCoVs were rare in stool samples from healthy children (0.7%, 95% CI 0–3.6%) and in NP swabs (1.9%, 95% CI 0.4–5.7%) is consistent with a report finding HCoVs in only 1.8% of stools from healthy children [11].

All four HCoVs species were detected in stool and NP samples; the most common was OC43 (50% in stool samples, 41% in NP samples). 229E occurred only in children with AGE without respiratory symptoms, whereas NL63 was found only in children with RTI as an additional sign.

## Conclusions

In conclusion, the presence of HCoVs in patients with AGE, the more frequent demonstration of these viruses in patients than in the control group and the higher probability of HCoVs detection in the stools of patients with AGE than in controls indicates some causal association of HCoVs with AGE. However, interpretation of the role of HCoVs in AGE is complex because the viruses were more often demonstrated in NP swabs than in stool samples, because of detection of other viruses known to cause AGE in stool samples and because more than half of the children with AGE had also signs and symptoms of RTI. The findings of the present study suggest that HCoVs probably played only a minor role in gastrointestinal illness in these children.

## Methods

### Study design

This was a part of a prospective study on viral respiratory and gastrointestinal infections in children <6 years. Children in this age group who had been admitted to the Department of Infectious Diseases of the University Medical Centre Ljubljana, from October 2009 to September 2011, with the diagnosis of AGE (defined as the passage of three or more loose or liquid stools in 24 h) were eligible for the study. Stool specimens, NP swabs and blood samples were obtained at admission and at a follow-up visit 14 days after the initial sampling. Children in the same age group admitted to the Department of Pediatric Surgery and Intensive Care for planned elective surgical procedures (i.e. inguinal hernia repair, testicular retention or hydrocele) were selected as a healthy control group. Only children without infections in last four weeks preceding the surgery were eligible for surgery admission. All children were clinically examined and only those without any symptoms of infection were admitted. Approximately one third of the admitted children have not been included due to parents’ refusal of entering the study or due to logistical problems with sample collection. In this group, stool samples and NP swabs were collected at admission. At this time, information was obtained on the presence of signs and symptoms compatible with gastrointestinal and/or respiratory infection within the last 14 days. To minimize children’s discomfort nasopharyngeal sampling was performed after patients underwent anesthesia.

The study protocol was approved by the National Medical Ethics Committee of the Republic of Slovenia (No. 87/08/09). Written informed consent was obtained from all parents of participating patients and control subjects. The principles of the Helsinki Declaration, the Oviedo Convention on Human Rights and Biomedicine and the Slovene Code of Medicine Deontology were strictly followed in the conduct of this research. The study protocol was also submitted to ClinicalTrials.gov registry with the title Viral respiratory and Gastrointestinal Infections in Children Under 6 Years of Age (reg. NCT00987519).

### Laboratory methods

Stool samples were diluted in sterile phosphate-buffered saline (PBS) to a 10% suspension. Aliquots of 180 μL of MagNA Pure bacteria lysis buffer (Roche Applied Science, Mannheim, Germany) and 20 μL of proteinase K (Qiagen, Hilden, Germany) were added to 190 μL of stool suspension.

NP swabs were collected using flocked-tip swabs and transported to the laboratory in the Copan universal transport medium (UTM-RT) system (Copan Italia, Brescia, Italy).

Before the extraction procedure, 5 μL of Equine herpesvirus 1 and 5 μL of Equine arthritis virus isolates were added to all samples. Specific target sequences of these viruses were subsequently amplified in separate real time reverse transcription polymerase chain reactions (RT–PCRs) as an internal control to ensure that negative results were not caused by poor nucleic acid extraction or inhibition of the RT–PCR assay [15,16].

The initial volume used for extracting total nucleic acids was 400 μL of stool suspension, 190 μL of vigorously vortexed NP swab medium and 190 μL of whole blood samples. Nucleic acids were extracted using total nucleic acid isolation kits on a MagNa Pure Compact instrument (Roche Applied Science), according to the manufacturer’s instructions.

Coronaviruses including HKU1, NL63, 229E and OC43 were detected in stool samples, NP swabs and whole blood samples by molecular methods using primers and probes as described by Kuypers et al. [17]. For amplification of 85–100 bp fragments of the polymerase 1b gene of coronaviruses, a one-step real-time RT–PCR assay was used in a StepOne Real-Time PCR system (Applied Biosystems, Carlsbad, CA). Briefly, 5 μL of total nucleic acid was added to 15 μL of reaction mixture including 2 × Reaction Mix, SuperScript^®^ III RT/Platinum^®^ Taq Mix (Invitrogen, Carlsbad, CA) with an additional 6 mM MgSO_4_. The cycling conditions were as follows: 20 min at 50°C, 2 min at 95°C and 45 cycles of 15 s at 95°C and 45 s at 60°C.

All NP swabs and stool samples in which HCoVs were established were also tested for the presence of other viruses. In NP swabs respiratory syncytial virus (RSV), influenza viruses A and B (Flu A-B), parainfluenza viruses 1–3 (PIV 1–3), metapneumovirus (hMPV), human bocavirus (HBoVs), adenovirus (AdV) and rhinovirus (hRV) [18-24] were searched for by real-time RT-PCR. Stool samples were tested for the presence of HBoVs and AdV using molecular methods as described previously [22,23], and for other gastroenteric viruses by electron microscopy (EM). The presence of norovirus genogroups I and II and astrovirus was ascertained by real-time RT-PCR [25,26], while group A rotavirus and adenovirus type 40/41 were detected by antigen-ELISA Premier Rotaclone and Premier Adenoclone (Meridian Bioscience, Cincinnati, OH).

### Statistical analysis

Data are reported as the frequency (percentage) and 95% confidence intervals (CIs) for proportions obtained using exact binomial tail areas. McNemar’s test was used to compare the frequency of HCoV positivity of patients at baseline and after 14 days of follow-up. Univariate logistic regression models with Firth’s correction were used to assess the association between HCoV positivity and study group (patient or control) and the results are reported as the odds ratio (OR) with 95% CI and two-sided *P* values. Mann–Whitney test was used to compare the median duration of illness and of hospitalization of HCoVs positive and HCoVs negative children.

## Abbreviations

HCoVs: Human coronaviruses; NP swab: Nasopharyngeal swab; RTI: Respiratory tract infections; AGE: Acute gastroenteritis.

## Competing interests

The authors declare that they have no competing interests. This study was supported by the Slovenian Research Agency (Research Program P3-0083) and institutional department funds.

## Authors’ contributions

MJ, MP^2^, TM, ŠG, FS and MP^1^ created the original idea of this research. MJ, AS, LL, TM, MP^2^, FS and MP^1^ designed the study and conducted analysis and interpretation of the data. TZ performed some of the laboratory methods. All authors read and approved the final manuscript.
